# Local Neuronal Synchronization in Frequent Nightmare Recallers and Healthy Controls: A Resting-State Functional Magnetic Resonance Imaging Study

**DOI:** 10.3389/fnins.2021.645255

**Published:** 2021-03-18

**Authors:** Louis-Philippe Marquis, Sarah-Hélène Julien, Véronique Daneault, Cloé Blanchette-Carrière, Tyna Paquette, Michelle Carr, Jean-Paul Soucy, Jacques Montplaisir, Tore Nielsen

**Affiliations:** ^1^Department of Psychology, Université de Montréal, Montreal, QC, Canada; ^2^Center for Advanced Research in Sleep Medicine, CIUSSS-NÎM – Hôpital du Sacré-Coeur de Montréal, Montreal, QC, Canada; ^3^Department of Psychiatry, Sleep & Neurophysiology Research Laboratory, University of Rochester Medical Center, Rochester, NY, United States; ^4^Montreal Neurological Institute, Montreal, QC, Canada; ^5^Department of Psychiatry, Université de Montréal, Montreal, QC, Canada

**Keywords:** nightmares, parasomnias, brain imaging, distress, psychopathology, regional homogeneity

## Abstract

Nightmares are highly dysphoric dreams that are well-remembered upon awakening. Frequent nightmares have been associated with psychopathology and emotional dysregulation, yet their neural mechanisms remain largely unknown. Our neurocognitive model posits that nightmares reflect dysfunction in a limbic-prefrontal circuit comprising medial prefrontal and anterior cingulate cortices, hippocampus, and amygdala. However, there is a paucity of studies that used brain imaging to directly test the neural correlates of nightmares. One such study compared the regional homogeneity (ReHo) of resting-state functional magnetic resonance imaging blood-oxygen level-dependent signals between frequent nightmare recallers and controls. The main results were greater regional homogeneity in the left anterior cingulate cortex and right inferior parietal lobule for the nightmare recallers than for the controls. In the present study, we aimed to document the ReHo correlates of frequent nightmares using several nightmare severity measures. We acquired resting-state functional magnetic resonance imaging data from 18 frequent nightmare recallers aged 18–35 (3 males and 15 females) and 18 age- and sex-matched controls, as well as retrospective and prospective disturbed dreaming frequency estimates and scores on the Nightmare Distress Questionnaire. While there were inconsistent results for our different analyses (group comparisons, correlational analyses for frequency estimates/Nightmare Distress scores), our results suggest that nightmares are associated with altered ReHo in frontal (medial prefrontal and inferior frontal), parietal, temporal and occipital regions, as well as some subcortical regions (thalamus). We also found a positive correlation between retrospective disturbed dreaming frequency estimates and ReHo values in the hippocampus. These findings are mostly in line with a recent SPECT study from our laboratory. Our results point to the possibility that a variety of regions, including but not limited to the limbic-prefrontal circuit of our neurocognitive model, contribute to nightmare formation.

## Introduction

Despite the clinical importance of diagnosing and treating nightmares (Gieselmann et al., 2019), the brain correlates of this enigmatic disorder are still largely unknown. Most studies of nightmare pathophysiology have focused on polysomnographically derived measures of sleep architecture (Germain and Nielsen, 2003; Nielsen et al., 2010b; Simor et al., 2012; Kis et al., 2014; Paul et al., 2015; Marquis et al., 2017; Blaskovich et al., 2019b), periodic leg movements (Germain and Nielsen, 2003), cardiac variability (Nielsen et al., 2010a; Simor et al., 2014; Perogamvros et al., 2019) or EEG metrics such as spectral power (Simor et al., 2013, 2014; Marquis et al., 2017; Blaskovich et al., 2019a), heartbeat-evoked potential (Perogamvros et al., 2019) and sleep spindle frequency and density (Nielsen et al., 2017, 2019; Picard-Deland et al., 2018a, b).

While such methods have provided useful clues to the neural mechanisms involved in nightmares, more direct brain imaging methods are beginning to identify implicated brain regions. Based on the findings of one study of a large cohort of patients with various types and localizations of brain damage, Solms (1997) proposed that some recurring nightmares are due to epileptiform activity in the temporal lobe. In contrast, a second study of 23 patients with bilateral calcification of the basolateral amygdala due to Urbach-Wiethe Disease (Blake et al., 2019) found that, compared to controls, patients with this lesion type have less frequent nightmares. Lesion studies therefore point to alterations in temporal regions and in the basolateral amygdala as contributors to nightmares.

There are still only a few research investigations of non-brain-lesioned patients with nightmares that employ brain imaging. All of these were realized during wakeful, resting-state conditions. Three of these were presented at conferences and/or published as abstracts by the same research group. One Suh et al. (2017) investigated resting-state seed-based functional connectivity in participants with Nightmare Disorder (*n* = 9) and healthy controls (*n* = 5) with a focus on posterior cingulate cortex, an important node of the Default Mode Network (Raichle et al., 2001). Compared to controls, nightmare participants had decreased functional connectivity in the right middle cingulate cortex and the medial frontal gyrus, and increased connectivity in the inferior frontal gyrus and parts of the cerebellum. A second Suh et al. (2018) investigated functional connectivity in 12 female Nightmare Disorder participants before and after 5 weekly sessions of Imagery Rehearsal Therapy, with the anterior cingulate cortex used as seed. Post-therapy, participants exhibited decreased connectivity between the anterior cingulate cortex and medial/orbitofrontal cortex and some parietal areas. Last, Park et al. (2019) compared the functional connectivity of Nightmare Disorder participants (*n* = 12) with that of an age- and sex-matched control group. Compared to controls, nightmare participants exhibited decreased connectivity between the posterior cingulate cortex and the left superior frontal gyrus. The results from these three investigations are quite inconsistent, possibly due to small sample sizes and the variable presence of trauma histories in the participants.

Three brain imaging investigations of nightmares have been published as journal articles. A recent single photon emission tomography (SPECT) study from our laboratory (Marquis et al., 2019a) showed that retrospectively measured dysphoric dream frequency and nightmare distress are negatively correlated with regional cerebral blood flow in several brain regions, but most consistently with blood flow in anterior cingulate cortex and medial prefrontal cortex. Additional findings from this study presented in conferences are that (1) these results are independent of psychopathology (Marquis et al., 2019b) and habitual dream recall frequency (Marquis et al., 2019c) and (2) multiple regression analyses suggest a lateralization pattern by which dysphoric dream frequency correlates primarily with left-hemisphere regions, whereas nightmare distress correlates primarily with right-hemisphere regions (Marquis et al., 2019d). Another recent study using near-infrared spectroscopy and a similar picture-viewing task during wakefulness, partially replicated past SPECT results for frontal regions (Carr et al., 2020b).

The third published study focused on Regional Homogeneity (ReHo) of functional magnetic resonance imaging (fMRI) blood oxygen level-dependent (BOLD) signals (Shen et al., 2016). ReHo is a measure of local neural synchronization obtained by computing Kendall’s concordance coefficient for each voxel and its (usually 26) nearest contiguous voxels (Zang et al., 2004). Shen et al. (2016) found that nightmare participants (*N* = 15) had, compared to the control group (*N* = 15), increased ReHo in the left anterior cingulate cortex and right inferior parietal lobule, and decreased ReHo in the left superior and inferior frontal gyri and bilateral middle occipital gyri. Additionally, the Physical Effect subscale of the Nightmare Experience Questionnaire (NEQ; Chen et al. (2014)), which measures “adverse effects of physical health, appetite, and other daily activities after nightmares,” was found to be positively correlated with ReHo values in the anterior cingulate cortex and inferior parietal lobule in the nightmare group (Shen et al., 2016). In the control group, however, this subscale was positively associated with ReHo values in the inferior frontal gyrus.

In sum, the neural correlates of nightmares remain largely unknown although lesion studies point to a role for temporal lobe regions and the basolateral amygdala in nightmare production. Existing brain imaging studies focusing on connectivity have (1) only been published as abstracts; (2) employed small sized samples; and (3) obtained inconsistent results but converge partially with two published brain imaging studies (Shen et al., 2016; Marquis et al., 2019a).

In light of the paucity of brain imaging research on nightmares, we aimed to document the ReHo correlates of nightmare-prone individuals using multiple nightmare severity measures. We compared ReHo of the BOLD signal for groups of frequent nightmare recallers and healthy controls. We also assessed whether there are graded relationships between scores on the Nightmare Distress Questionnaire and ReHo values in both groups. Finally, we assessed relationships between retrospective and prospective disturbed dreaming frequencies and ReHo values.

## Materials and Methods

### Participants

Participants were recruited as part of a larger brain imaging study of frequent nightmare recallers. Our sample overlaps with that of a previous study in our laboratory (Marquis et al., 2019a), for which preliminary findings were published in abstract form (Marquis et al., 2016). The reader is referred to this study for details on recruitment methods, screening interviews, inclusion criteria, etc. The study was approved by institutional ethics and scientific committees. Participants signed a consent form containing a full description of the study protocol during their first visit to the laboratory. They were compensated for their time and transport expenses.

Briefly, we recruited French and English-speaking participants aged 18 to 35 through advertisements on local university campuses, our laboratory’s website and by word of mouth. A screening interview was conducted to verify that participants had at least two nightmares and/or bad dreams (dysphoric dreams without awakening) per week for the nightmare (NM) group, or less than one per month for the control (CTL) group, as well as to ascertain that they met the other inclusion criteria. Based on participants’ responses to the screening interview, our sample was free from comorbid sleep disorders and from psychiatric or medical conditions susceptible to affect dreaming and brain imaging results. Participants reported taking less than 10 alcoholic beverages per week and not using drugs except marijuana (1/month or less). They took no medications other than oral contraceptives. They also did not report any traumatic events in the past 6 months.

From our initial sample, we excluded two NM participants who reported a traumatic event on the Posttraumatic Stress Disorder Checklist and scored over the recommended cut-off point. One CTL participant was moderately depressed according to the Beck Depression Inventory-II cut-off and was excluded from further analyses. One NM participant scored above the cut-offs for both questionnaires. Another NM participant had an abnormality on structural imaging. After excluding these participants, we age- and sex-matched our NM and CTL participants. The final sample included 18 frequent NM recallers (3 males and 15 females) and 18 controls (3 males and 15 females). Participants were right-handed except for one in the NM group and two in the CTL group. Sample characteristics are shown in Table 1.

**TABLE 1:** Participant characteristics.

	CTL group		NM group			
Measures	*M*	*SD*	*M*	*SD*	*p*	*t*
Age (years)	24.01	4.92	25.50	4.82	>0.10	
Sex (M:F)	3:15		3 :15			
Education level-Highest level completed (n)						
-High school	2 (1 studying^a^)		3 (2 studying^a^)			
-Professional diploma	3 (1 studying^a^)		1			
-Pre-university diploma	6 (5 studying^a^)		5 (4 studying^a^)			
-Bachelor’s degree	5 (4 studying^a^)		7 (1 studying^a^)			
-Graduate degree	2		2 (1 studying^a^)			
BDI-II (raw score)	5.50	4.74	5.78	4.69	>0.10	
NDQ (raw score)	24.67	5.01	32.06	6.74	*=0.001*	3.61
STAI-Trait (raw score)	33.06	8.73	32.00	9.73	>0.10	
STAI-State (raw score)	29.22	6.04	29.17	5.87	>0.10	
**Retrospective recall^b^**						
Dreams (#/week)*^d^*	2.81	1.49	6.14	2.21	*<0.001*	5.33
Bad dreams (#/week)*^d^*	0.17	0.15	2.53	1.30	*<0.001*	10.37
Nightmares (#/week)*^d^*	0.08	0.18	0.97	1.05	*<0.001*	4.42
Dysphoric dreams (#/week)*^d^*	0.22	0.20	3.49	1.64	*<0.001*	15.15
**Prospective recall^c^**						
Dreams (#/week)	2.10	1.64	1.83	1.08	>0.10	
Bad dreams (#/week)	1.07	0.98	2.20	1.41	*=0.009*	2.79
Nightmares (#/week)*^d^*	0.25	0.39	1.11	1.02	*=0.001*	3.65
Dysphoric dreams (#/week)	1.32	1.11	3.31	1.23	*<0.001*	5.10

*Regarding education level: Please note that Québec has a 5-year high school degree and a 2-year pre-university degree (CÉGEP). The p-values are the result of independent samples t-tests; values less than 0.06 are italicized.*

*^a^Number of participants studying for higher degree.*

*^b^Retrospective measures from screening interview.*

*^c^Prospective measures from sleep-dream log.*

*^d^Variables log-transformed (value+1) for statistical analysis.*

*STAI-Trait, State-Trait Anxiety Inventory-Trait; STAI-State, State-Trait Anxiety Inventory-State; BDI-II, Beck Depression Inventory-II; NDQ, Nightmare Distress Questionnaire.*

### Procedure

During the laboratory visit, participants completed questionnaires including those listed in the following section. While each participant’s MRI compatibility was assessed during the screening interview, it was formally assessed during this visit in compliance with MRI safety guidelines. Participants were then instructed on how to complete each part of the study, including a 2-week home sleep-dream log starting the following morning. If eligible, participants returned to the laboratory 1 and 2 weeks later for 2 SPECT scans (see Marquis et al. (2019a)). Participants typically underwent an MRI scan within 1 month of their visit to the laboratory, according to staff and participant availability. MRI scans were performed at the Functional Neuroimaging Unit of the Montréal University Institute of Geriatrics Research Center. During the resting-state sequences, participants were instructed to keep their eyes open, to look at a fixation cross, and to not move or think about anything. They were also asked not to fall asleep. Participants’ faces were filmed during all scanning sequences so the research team could verify their compliance to with instructions.

### Questionnaires

To better characterize our sample and to ensure that participants met the inclusion criteria, they completed the State-Trait Anxiety Inventory [STAI; Spielberger and Gorsuch (1983)], the Beck Depression Inventory-II [BDI-II; Beck et al. (1996)], the Posttraumatic Stress Disorder Checklist for DSM-5 [PCL-5; Weathers et al. (2013)] and the Nightmare Distress Questionnaire [NDQ; Belicki (1992)]. A cut-off exclusion score of >19 was used for the BDI-II, corresponding to moderate depression (Dozois et al., 1998). This liberal cut-off aims to account for the frequently, but not always, observed link between depression and nightmare frequency/distress (Zadra and Donderi, 2000; Blagrove et al., 2004; Miro and Martinez, 2005; Blagrove and Fisher, 2009). One participant from the CTL group and one from the NM group each had BDI-II scores between 14 and 19, values considered to indicate mild depression (Dozois et al., 1998). Questionnaire results are shown in Table 1.

#### Home Sleep-Dream Log

Each participant completed a 2-week home sleep-dream log using a Voicemail Interactive System as in previous research from our laboratory (Dumel et al., 2015; Marquis et al., 2019a). It assessed various dream properties, mostly on 1–9 scales (0 if there was no dream recall). This sleep-dream log was used to obtain prospective estimates of dream recall (when recall clarity was ≥1 out of 9), bad dream recall (when negative emotion was ≥5) and nightmare recall (when negative emotion was ≥5 and the dream caused an awakening). Prospective estimates for dysphoric dream recall were obtained by combining bad dream and nightmare measures. All of this information is shown in Table 1.

#### Retrospective Measures

As in previous research (Marquis et al., 2019a), retrospective measures were obtained from the initial telephone screening interview and computed as weekly frequencies of recalling dreams, bad dreams, nightmares and dysphoric dreams (see Table 1).

### MRI Acquisition

We used a 3.0 Tesla Siemens TrioTim MRI scanner. During our recruitment period, the scanner was upgraded to a 3.0 Tesla Siemens Prisma Fit. Thus, 28 participants were scanned using the TrioTim, and 4 participants using the Prisma Fit. While the MRI sequences are equivalent for the two devices, scanner model was entered as a nuisance variable in all statistical analyses for brain imaging data. Acquisitions all occurred between 9:00 and 17:00 according to participant preference and staff availability.

Functional magnetic resonance imaging BOLD signal was acquired by single-shot gradient-echo echo-planar imaging sequence. For each participant, we collected 150 contiguous functional volumes during resting-state in approximately 6.5 min with the following parameters: repetition time = 2600 ms; echo time = 30 ms; flip angle = 90°, field of view = 218 mm × 218 mm; matrix size = 64 × 64; number of slices = 42; and slice thickness was 3.4 mm without gap. The sequences were equivalent for our two scanner models. A T1-weighted anatomical image was also acquired in approximately 6 min by a multi-echo magnetization-prepared rapid gradient echo sequence with the following parameters: field of view = 256 mm × 256 mm; matrix size = 256 × 256; 176 sagittal slices; resolution = 1-mm isotropic; repetition time = 2530 ms/root mean square of 4 echo times = 1.64 ms, 3.5 ms, 5.36 ms, 7.22 ms; and flip angle = 7°. For the Prisma Fit scanner, the T1 weighted-sequence had the following parameters: field of view = 256 mm × 256 mm; matrix size = 256 × 256; 176 sagittal slices; resolution = 1-mm isotropic; repetition time = 2090 ms/root mean square of 4 echo times = 1.69 ms, 3.55 ms, 5.41 ms, 7.27 ms; and flip angle = 8°. Additional details about the T1 sequence can be found elsewhere (Van Der Kouwe et al., 2008).

### fMRI Preprocessing

Functional image preprocessing was conducted using the Data Processing and Analysis for (Resting-State) Brain Imaging (DPABI; Yan et al. (2016)]^1^. For each participant, we removed the first 10 functional volumes. We then performed slice timing and head motion correction. The head motion profile (six-dimensional; three for translation and three for rotation) for each participant was estimated. No participant had a translation more than 1.5 mm in any cardinal direction or a rotation more than 1.5° in any axis. The realigned functional images were then spatially normalized to the Montreal Neurological Institute space using the normalization parameters estimated by T1 structural image (after brain extraction; see Smith (2002)) segmented with the Diffeomorphic Anatomical Registration using Exponentiated Lie algebra (DARTEL) algorithm (Ashburner, 2007), re-sampled to 3 mm × 3 mm × 3 mm voxels. We regressed out nuisance variables, namely motion parameters [using Friston’s 24-parameter model; Friston et al. (1996)], average white matter, and cerebrospinal fluid signals. Following this, the fMRI data were linearly de-trended and temporally band-pass filtered (0.01–0.08 Hz).

### ReHo Calculation

We generated individual ReHo maps by computing Kendall’s concordance coefficient [KCC; Kendall and Gibbons (1990)]. KCC measures ReHo of BOLD time series for each voxel and the nearest 26 contiguous voxels (Zang et al., 2004) and is calculated as follows:

$$W=\frac{\Sigma \,{\left(R_{i}\right)}^{2}-n\,{\left(\bar{R}\right)}^{2}}{\frac{1}{12}\,K^{2}\,\left(n^{3}-n\right)}$$

“where *W* is the KCC among given voxels, ranged from 0 to 1; *R*_*i*_ is the sum rank of the *i*th time point; where $\bar{R}$ = ((*n* + 1) *K*)/2 is the mean of the *R*_*i*_’s; *K* is the number of time series within a measured cluster [(…) *K* = 27, one given voxel plus the number of its neighbors]; *n* is the number of ranks (…) (Zang et al., 2004).”

For every participant, the KCC map was then normalized by dividing KCC in each voxel by the mean KCC of total gray matter. Finally, the ReHo maps were smoothed using a 4-mm full-width at half-maximum (FWHM) Gaussian kernel. All these steps were accomplished using DPABI and the built-in gray matter mask.

### Statistical Analyses

#### Demographics, Questionnaires, Screening Interview, Home Sleep-Dream Log

Distributions of these measures were examined for normality and descriptive statistics generated with SPSS 26 (IBM Inc., Armonk, United States). The groups were compared on relevant variables using independent sample *t*-tests with a statistical threshold of *p* < 0.05 (two-tailed).

#### ReHo Analyses

To evaluate group differences in ReHo, we used multiple *t*-tests for independent samples with a corrected significance threshold of *p* < 0.05 (two-tailed) by combining thresholds of *p* < 0.01 at voxel-level and *k* > 26 at cluster-level. ReHo analyses controlled for age, sex, and scanner model. This cluster extent threshold is based on calculations in AlphaSim as implemented in Rest software v1.8 Song et al. (2011); 5000 Monte Carlo iterations, FWHM = 4 mm). Our analyses are based on whole-brain data.

We also correlated our nightmare severity measures with ReHo values using the same statistical threshold, namely (1) scores on the Nightmare Distress Questionnaire (separately for each group), (2) retrospective disturbed dreaming frequency estimates (NM group only), and (3) prospective disturbed dreaming frequency estimates (NM group only).

Statistical analyses were performed using SPM12 Friston et al. (1994); Statistical Parametric Mapping 12, Wellcome Trust Centre for Neuroimaging, Institute of Neurology, University College London, United Kingdom) with MatLab (ver9.4, The Mathworks, Natick, MA, United States). Analyses were performed for each voxel of gray matter using a gray matter mask. The mask was generated as part of the DPABI preprocessing pipeline (Yan et al., 2016).

Significant regions were identified using the ICBM atlas (Mazziotta et al., 2001) in the PickAtlas software (version 3.0.5; Maldjian et al. (2003)). We used the MRIcron program^2^ to generate figures.

## Results

### Demographic, Questionnaires, Screening Interview, Sleep-Dream Log

Means and standard deviations for these variables are shown in Table 1, as well as results from group comparisons. Briefly, our groups did not differ on age, sex ratio, BDI-II and STAI scores, and prospective dream recall frequency (*p* > 0.10). Compared to the CTL group, the NM group had a lower retrospective dream recall frequency, higher NDQ scores and higher frequencies of bad dream, nightmare and dysphoric dream recall for both types of estimates (retrospective and prospective; all *p* < 0.05).

### ReHo Analyses

**FIGURE 1:**
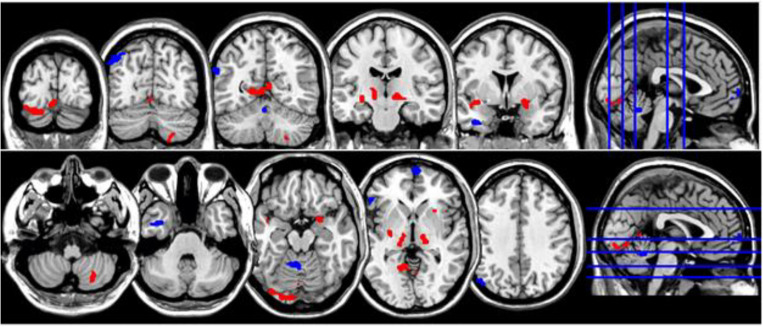
Coronal and axial multislice views of regions with significant group differences in ReHo values. Color code; red, regions for which the NM group exhibited lower ReHo values than did controls; blue, regions for which the NM group exhibited higher ReHo values than did controls. Significant regions were obtained with the following combination of statistical thresholds: *p* < 0.01 at voxel level within clusters >26.

**FIGURE 2:**
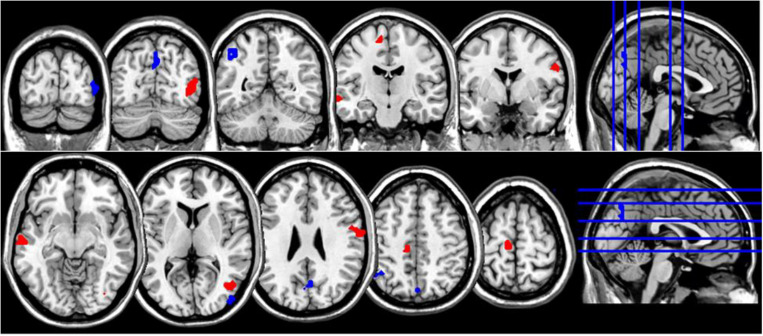
Coronal and axial multislice views of regions showing correlations between NDQ scores and ReHo values in NM group. Color code; red, regions for which the NDQ scores were associated with increased ReHo values; blue, regions for which the NDQ scores were associated with decreased ReHo values. Significant regions were obtained with the following combination of statistical thresholds: *p* < 0.01 at voxel level within clusters >26.

**FIGURE 3:**
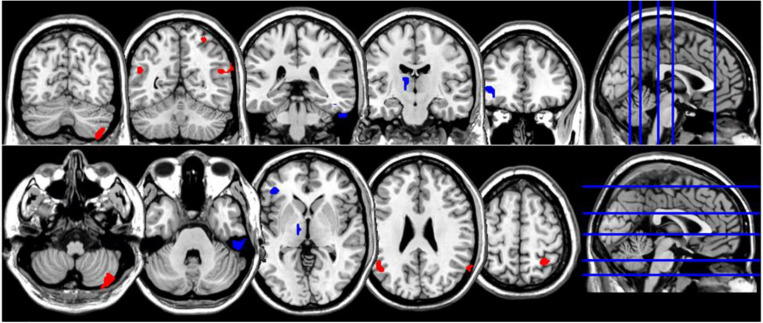
Coronal and axial multislice views of regions showing correlations between NDQ scores and ReHo values in CTL group. Color code; red, regions for which the NDQ scores were associated with increased ReHo values; blue, regions for which the NDQ scores were associated with decreased ReHo values. Significant regions were obtained with the following combination of statistical thresholds: *p* < 0.01 at voxel level within clusters >26.

**FIGURE 4:**
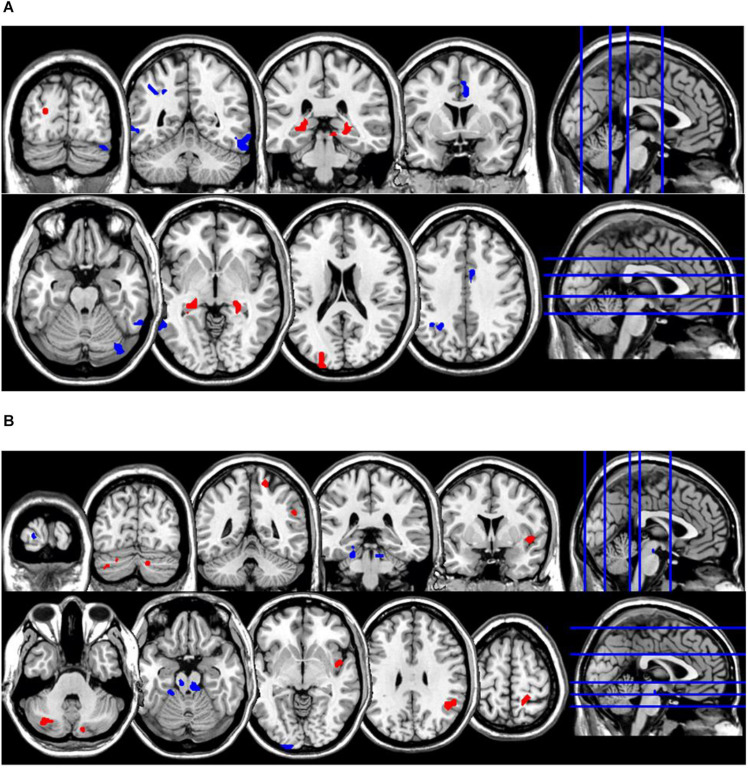
Coronal and axial multislice views of regions showing correlations between ReHo values in the NM group and estimates of **(A)** retrospective disturbed dreaming frequency and **(B)** prospective disturbed dreaming frequency. Color code; red, regions for which the retrospective disturbed dreaming frequency estimates were associated with increased ReHo values; blue, regions for which the retrospective disturbed dreaming frequency estimates were associated with decreased ReHo values. Significant regions were obtained with the following combination of statistical thresholds: *p* < 0.01 at voxel level within clusters >26.

There were group differences in ReHo values for both contrasts (CTL < NM and CTL > NM). All relevant information (cluster size, peak locations, *p*- and *t*-values, hemisphere, Brodmann area equivalent, and MNI coordinates X, Y, and Z) is listed in Table 2 and Figure 1. In the NM group, we observed positive and negative correlations between NDQ scores and ReHo values. All relevant information is reported in Table 3 and Figure 2. Results from similar correlational analyses for the CTL group are reported in Table 4 and Figure 3. We also observed positive and negative correlations between estimates of retrospective and prospective dysphoric dreaming recall and ReHo values in the NM group (reported, respectively, in Tables 5, 6 and Figure 4). Table 7 is provided to facilitate easy comparison of major findings between analyses.

**TABLE 2:** Localization of group differences in ReHo values.

Cluster size (k)	Location	*P*			Peak	MNI coordinates		
			Side	BA	*t*-values	*x*	*y*	*z*
**NM group < CTL group (decreased ReHo)**								
67	Putamen	<0.001	L	–	4.99	−30	−15	−6
	Putamen	<0.001	L	–	3.42	−33	0	−9
	Superior temporal cortex	<0.001	L	13	3.19	−42	0	−15
44	Putamen	<0.001	L	–	3.87	−21	15	−9
66	Subcallosal Gyrus	<0.001	R	34	3.94	24	6	−15
	Putamen	<0.001	R	–	3.81	24	12	−6
	Putamen	=0.005	R	–	2.73	27	18	0
147	Fusiform gyrus	<0.001	L	19	4.45	−24	−87	−21
	Inferior occipital gyrus	<0.001	L	18	4.10	−36	−84	−18
	Lingual gyrus	<0.001	L	17	3.93	−9	−93	−15
72	Posterior cingulate gyrus	<0.001	L	30	3.88	−6	−57	3
	Posterior cingulate gyrus	<0.001	L	30	3.55	−18	−60	6
	Posterior cingulate gyrus	<0.005	R	29	3.17	3	−57	9
42	Thalamus	<0.001	L	–	3.76	−12	−15	−3
	Thalamus	<0.001	L	–	3.62	−15	−27	−3
54	Thalamus	<0.001	R	–	4.61	15	−18	−3
	Thalamus	<0.001	R	–	3.02	18	−24	6
39	Cerebellum	<0.001	R	–	3.55	27	−66	−51
	Cerebellum	=0.005	R	–	2.72	27	−54	−57
**NM group > CTL group (increased ReHo)**								
42	Cerebellum	<0.001	L	–	4.82	−6	−48	−18
	Cerebellum	<0.001	L	–	3.63	−18	−51	−21
88	Precuneus	<0.001	L	19	4.00	−42	−75	42
	Supramarginal gyrus	<0.001	L	40	3.63	−60	−63	30
50	Medial frontal gyrus	<0.001	R	10	3.68	3	69	0
	Medial frontal gyrus	=0.005	L	10	2.70	−6	60	−3
49	Inferior frontal gyrus	<0.005	L	47	3.33	−45	24	−6
	Inferior frontal gyrus	<0.005	L	47	3.29	−54	21	−9
	Inferior frontal gyrus	<0.005	L	47	2.97	−54	27	−3
34	Inferior temporal gyrus	<0.005	L	21	3.29	−48	0	−33
	^b^	<0.005	L	^a^	3.20	−36	0	−33
	^b^	<0.005	L	^a^	3.10	−42	−6	−24

*MNI, Montreal Neurological Institute; BA, Brodmann area; L, left; R, right. Significant regions were obtained with the following combination of statistical thresholds: peaks at p < 0.01 at the voxel level within clusters >26.*

*^a^PickAtlas Software was unable to give BA equivalent.*

*^b^PickAtlas Software was unable to give location.*

**TABLE 3:** Localization of regions showing correlations between NDQ scores and ReHo values in NM group.

Cluster size (k)	Location	*P*			Peak	MNI coordinates		
			Side	BA	*t*-values	*x*	*y*	*z*
**Regions associated with increased ReHo**								
34	Middle temporal gyrus	<0.001	L	21	7.05	−63	−15	−9
97	Middle occipital gyrus	<0.001	R	19	4.93	42	−72	0
	Middle temporal gyrus	<0.005	R	37	3.76	51	−69	12
64	Medial frontal gyrus	<0.001	L	6	4.64	−9	−21	66
	Medial frontal gyrus	<0.001	L	6	4.50	−12	−24	51
64	Precentral gyrus	<0.001	R	6	4.76	63	−6	27
	Postcentral gyrus	<0.001	R	43	3.86	63	−9	18
	Inferior frontal gyrus	=0.005	R	9	2.96	51	3	24
**Regions associated with decreased ReHo**								
49	Precuneus	<0.001	R	7	4.79	3	−72	45
	Precuneus	<0.001	R	31	4.01	6	−66	27
	Precuneus	<0.001	R	7	3.93	0	−72	33
36	Inferior parietal lobule	<0.001	L	40	4.28	−39	−51	45
	Inferior parietal lobule	<0.005	L	40	3.46	−48	−54	39
	Inferior parietal lobule	<0.005	L	40	3.39	−51	−57	51
38	Middle occipital gyrus	=0.001	R	19	3.73	42	−87	12

*MNI, Montreal Neurological Institute; BA, Brodmann area; L, left; R, right. Significant regions were obtained with the following combination of statistical thresholds: peaks at *p* < 0.01 at the voxel level within clusters >26.*

**TABLE 4:** Localization of regions showing correlations between NDQ scores and ReHo values in the CTL group.

Cluster size (k)	Location	*P*			Peak	MNI coordinates		
			Side	BA	*t*-values	*x*	*y*	*z*
**Regions associated with increased ReHo**								
29	Middle temporal gyrus	<0.001	L	39	4.82	−48	−60	24
56	Superior parietal lobule	<0.001	R	7	4.67	30	−54	63
	Precuneus	<0.001	R	7	4.17	27	−51	54
	Inferior parietal lobule	<0.005	R	40	3.63	39	−48	54
28	Superior temporal gyrus	<0.001	R	39	4.21	63	−60	24
	Supramarginal gyrus	<0.005	R	39	3.72	51	−57	21
61	Cerebellum	<0.001	R	–	4.20	39	−81	−51
	Cerebellum	<0.005	R	–	3.45	39	−69	−54
	Cerebellum	<0.005	R	–	3.13	48	−75	−48
**Regions associated with decreased ReHo**								
33	Thalamus	<0.001	L	–	5.00	−12	−15	12
	Thalamus	<0.001	L	–	4.13	−12	−12	0
	Lentiform nucleus	<0.005	L	–	3.29	−12	−3	−3
30	Inferior frontal gyrus	<0.001	L	^*a*^	4.52	−42	36	0
	Middle frontal gyrus	<0.005	L	11	3.43	−30	45	−6
42	Fusiform gyrus	<0.001	R	20	3.86	54	−33	−33
	Fusiform gyrus	<0.005	R	20	3.19	45	−30	−21

*MNI, Montreal Neurological Institute; BA, Brodmann area; L, left; R, right. Significant regions were obtained with the following combination of statistical thresholds: peaks at p < 0.01 at the voxel level within clusters >26.*

*^a^PickAtlas Software was unable to give BA equivalent.*

**TABLE 5:** Localization of regions showing correlations between retrospective disturbed dreaming frequency estimates and ReHo values in the NM group.

Cluster size (k)	Location	*P*			Peak	MNI coordinates		
			Side	BA	*t*-values	*x*	*y*	*z*
**Regions associated with increased ReHo**								
51	Thalamus	<0.001	L	–	7.72	−24	−24	−6
57	Hippocampus	<0.001	R	–	5.08	21	−27	−9
	Hippocampus	<0.005	R	–	3.37	27	−36	0
	Parahippocampal gyrus	<0.005	R	–	3.34	12	−33	−6
39	Cuneus	<0.001	L	19	4.37	−24	−93	21
**Regions associated with decreased ReHo**								
27	Inferior parietal lobule	<0.001	L	40	6.06	−27	−54	39
	Inferior parietal lobule	<0.005	L	40	3.59	−39	−51	42
38	Middle temporal gyrus	<0.001	L	21	5.13	−63	−54	0
	Middle temporal gyrus	<0.005	L	21	3.10	−54	−48	−6
50	Middle temporal gyrus	<0.005	R	21	4.19	57	−51	−15
	Middle temporal gyrus	=0.005	R	21	2.99	63	−42	−9
33	Cingulate gyrus	<0.001	R	24	4.02	6	6	36
	Medial frontal gyrus	<0.005	R	32	3.49	3	3	48
	Cingulate gyrus	<0.005	R	24	3.20	6	−6	42
40	Cerebellum	<0.005	R	–	3.70	39	−78	−24
	Cerebellum	<0.005	R	–	3.54	51	−75	−27
	Fusiform gyrus	<0.005	R	19	3.28	27	−78	−18

*MNI, Montreal Neurological Institute; BA, Brodmann area; L, left; R, right. Significant regions were obtained with the following combination of statistical thresholds: peaks at *p* < 0.01 at the voxel level within clusters >26.*

**TABLE 6:** Localization of regions showing correlations between prospective disturbed dreaming frequency estimates and ReHo values in the NM group.

Cluster size (k)	Location	*P*			Peak	MNI coordinates		
			Side	BA	*t*-values	*x*	*y*	*z*
**Regions associated with increased ReHo**								
27	Superior temporal gyrus	<0.001	R	22	6.12	48	3	−3
43	Supramarginal gyrus	<0.001	R	40	5.84	54	−48	30
	Supramarginal gyrus	<0.001	R	*^*a*^*	4.28	42	−51	30
	Inferior parietal lobule	<0.005	R	40	3.36	48	−54	42
29	Postcentral gyrus	<0.001	R	*^*a*^*	5.17	18	−45	66
	Superior parietal lobule	<0.005	R	*^*a*^*	3.12	15	−51	60
41	Cerebellum	<0.005	L	–	3.77	−36	−72	−36
	Cerebellum	<0.005	L	–	3.62	−27	−72	−36
	Cerebellum	<0.005	L	–	3.18	−21	−78	−27
32	Cerebellum	<0.005	R	–	3.35	12	−78	−33
	Cerebellum	=0.005	R	–	2.97	12	−87	−27
**Regions associated with decreased ReHo**								
66	Cerebellum	<0.001	R	–	6.03	9	−30	−21
	Brain stem	<0.001	L	–	3.95	−9	−24	−18
	*^*b*^*	<0.005	L	–	3.22	−3	−18	−18
29	Cerebellum	<0.001	L	–	4.71	−21	−36	−21
	Parahippocampal gyrus	<0.005	L	–	3.82	−21	−36	−12
39	Cuneus	<0.001	L	18	4.17	−21	−102	−6

*MNI, Montreal Neurological Institute; BA, Brodmann area; L, left; R, right. Significant regions were obtained with the following combination of statistical thresholds: peaks at p < 0.01 at the voxel level within clusters >26.*

*^a^PickAtlas Software was unable to give BA equivalent.*

*^b^Pickatlas Software was unable to give location.*

**TABLE 7:** Summary of regions showing group differences in ReHo values and/or a correlation with ReHo values.

#2. Group differences in ReHo values				#3. NDQ-ReHo correlation in NM group				#4. NDQ-ReHo correlation in CTL group				#5. RetroDDF-ReHo correlation in NM group				#6. ProsDDF-ReHo correlation in NM group			

Location	Side	BA	Effect	Location	Side	BA	Effect	Location	Side	BA	Effect	Location	Side	BA	Effect	Location	Side	BA	Effect
**Temporal**																			
Inferior temporal gyrus	L	21	NM > CTL																
				Middle temporal gyrus	L	21	+	Middle temporal gyrus	L	39	+	Middle temporal gyrus	L/R	21	−				
				Middle temporal gyrus	R	37	+												
Superior temporal cortex	L	13	NM < CTL					Superior temporal gyrus	R	39	+					Superior temporal gyrus	R	22	+
**Occipital**																			
Fusiform gyrus	L	19	NM < CTL					Fusiform gyrus	R	20	−	Fusiform gyrus	R	19	−				
Inferior occipital gyrus	L	18	NM < CTL																
				Middle occipital gyrus	R	19	−												
				Middle occipital gyrus	R	19	+												
Lingual gyrus	L	17	NM < CTL																
Precuneus	L	19	NM > CTL	Precuneus	R	7	−	Precuneus	R	7	+								
												Cuneus	L	19	+	Cuneus	L	18	−
**Parietal**																			
Supramarginal gyrus	L	40	NM > CTL					Supramarginal gyrus	R	39	+					Supramarginal gyrus	R	40	+
				Precentral gyrus	R	6	+												
				Postcentral gyrus	R	43	+									Postcentral gyrus	R		+
				Inferior parietal lobule	L	40	−	Inferior parietal lobule	R	40	+	Inferior parietal lobule	L	40	−	Inferior parietal lobule	R	40	+
								Superior parietal lobule	R	7	+					Superior parietal lobule	R		+
**Frontal**																			
Medial frontal gyrus	L/R	10	NM > CTL	Medial frontal gyrus	L	6	+					Medial frontal gyrus	R	32	−				
Inferior frontal gyrus	L	47	NM > CTL	Inferior frontal gyrus	R	9	+	Inferior frontal gyrus	L	^*a*^	−								
								Middle frontal gyrus	L	11	−								
**Cingulate**																			
Subcallosal Gyrus	R	34	NM < CTL																
Posterior cingulate gyrus	L/R	30	NM < CTL																
												Cingulate gyrus	R	24	−				
**Subcortical**																			
Putamen	L/R	−	NM < CTL																
Thalamus	L/R	−	NM < CTL					Thalamus/Lentiform nucleus	L	−	−	Thalamus	L	−	+				
Cerebellum	L	−	NM > CTL													Cerebellum	L/R	−	+
Cerebellum	R	−	NM < CTL					Cerebellum	R	−	+	Cerebellum	R	−	−	Cerebellum	L	−	−
																Cerebellum	R	−	−
												Hippocampus	R	−	+				
												Parahippocampal gyrus	R	−	+	Parahippocampal gyrus	L	−	−
																Brain stem	L	−	−

*Similar results are aligned to show similarities between analyses. BA, Brodmann area; L, left; R, right. Effect: result of group comparisons or direction of correlation; +, positive; −, negative. Significant regions were obtained with the following combination of statistical thresholds: peaks at *p* < 0.01 at the voxel level within clusters >26.*

*^*a*^PickAtlas Software was unable to give BA equivalent.*

## Discussion

Our analyses yielded several significant findings. For simplicity, in the following section, we label the analyses according to their corresponding table number. Therefore, #2 corresponds to group comparisons in Table 2, #3 corresponds to NDQ-ReHo correlations for the NM group in Table 3, #4 the same correlations as in #3 but for the CTL group (Table 4), #5 corresponds to correlations between retrospective disturbed dreaming frequency estimates in Table 5 and #6 corresponds to correlations between prospective disturbed dreaming frequency estimates in Table 6. The following summarizes central results that (a) are featured in multiple analyses, (b) have a special theoretical relevance, or (c) are relevant in the context of previous empirical research on the correlates of NMs.

Notable results are found in frontal regions, namely the medial prefrontal gyrus (increased ReHo for analyses #2 and #3, decreased ReHo for analysis #5) and the inferior frontal gyrus (increased ReHo for analyses #2–#3, decreased ReHo for analysis #4). Surprisingly, results involving the anterior cingulate gyrus were only found in analysis #5 (decreased ReHo/negative correlation). The middle temporal gyrus was featured in analyses #3 and #4 (increased ReHo) and #5 (decreased ReHo); the results involving other temporal areas were inconsistent. Among occipital regions, the fusiform gyrus was consistently featured (increased ReHo) in analyses #2, #4 and #5 while the precuneus was featured in analyses #2 and #4 (increased ReHo) and #3 (decreased ReHo). For parietal regions, the supramarginal gyrus was featured in analyses #2, #4 and #6 (increased ReHo) and the inferior parietal lobule was featured in analyses #3 and #5 (decreased ReHo) and #4 and #6 (increased ReHo). Among subcortical regions, the thalamus was featured in analyses #2 and #4 (decreased ReHo) and #5 (increased ReHo). The hippocampus was featured in analysis #5 (increased ReHo). Finally, the cerebellum was featured in analyses #2, #5 and #6 (decreased ReHo) and #4 (increased ReHo). In sum, we found nightmare severity to be associated with altered ReHo in various frontal, temporal, occipital and parietal regions.

As is evident from this summary, there are inconsistent results between analyses. However, some of these inconsistencies may have theoretical relevance. For example, inconsistencies between analyses #3 and #4 may indicate brain correlates of nightmare distress that are unique to frequent NM sufferers. Such results could challenge the hypothesis of shared brain structures involved in all disturbed dreaming/nightmares (Levin and Nielsen, 2007), but more work will be necessary to test this hypothesis. Inconsistencies between analyses #3 and #5 could be the result of different neural correlates underlying NM frequency and NM distress. Limited evidence points to only partially shared brain correlates for the two variables (Marquis et al., 2019a).

One of our findings is consistent with those of published results that also used a ReHo approach (Shen et al., 2016). We found a negative correlation between ReHo values in the inferior parietal lobule and NDQ scores in the NM group: Shen et al. obtained a similar result using a Nightmare Experience Questionnaire subscale.

However, apart from this similarity our findings differed from published results (Shen et al., 2016). We did not find group differences for ReHo values in the anterior cingulate cortex nor in the inferior parietal lobule. We found that frequent nightmare recallers have increased ReHo values in the inferior frontal gyrus, while Shen et al. (2016) found the opposite result. Shen et al. observed a positive correlation between the NEQ Physical Effect subscale and the ReHo values in the inferior frontal gyrus (CTL group only), while in our CTL group we observed a negative correlation between NDQ scores and ReHo values in the same region.

We propose that three main factors contribute to the discrepancies between our results and those from this past study (Shen et al., 2016). First, for the correlational analyses, the two studies used a different questionnaire measure (the NDQ and NEQ). The two questionnaires measure similar but not identical constructs, and there is no study comparing these measures. Second, the subject pools of NM sufferers are different and, arguably, not comparable. While our NM participants experienced more frequent NMs than did those of Shen et al., the level of nightmare distress of their group is unclear. They diagnosed their participants with DSM-5 criteria (American Psychiatric Association, 2013), which is very different from measuring levels of nightmare distress. Clinically, nightmare distress is considered more important than nightmare frequency. Third, their control group was recruited with very stringent criterion (0–1 nightmares per lifetime). This method of selecting participants may have increased the contrast between groups. As suggested by a reviewer, one way to reconcile results from the two studies is to propose different neural correlates for different levels of nightmare severity. This possibility should be addressed in future work because, as indicated earlier, the study cohorts are arguably not comparable.

Our results are more in line with a recent SPECT study from our laboratory (Marquis et al., 2019a). While not present in all our analyses, both studies highlight the possible role of the anterior cingulate cortex and medial prefrontal cortex. Both studies also have found nightmares to be associated with cortical regions involved in sensory processing (parietal, occipital, and temporal cortices). Finally, both studies produced stronger results for retrospective than for prospective disturbed dreaming estimates.

Our neurocognitive model of nightmares (Levin and Nielsen, 2007; Nielsen and Levin, 2007) proposes that nightmares are produced mainly by dysfunction in a limbic-prefrontal circuit comprising the amygdala, hippocampus, anterior cingulate cortex and medial prefrontal cortex. As stated earlier, the limited brain imaging literature on nightmares mainly supports the possible involvement of the anterior cingulate and medial prefrontal cortex. In a previous article by our group, these results were interpreted primarily as evidence of a cross-state alteration of emotional processing. While our neurocognitive model emphasizes a role for these structures in emotion regulation, other research also suggests roles in emotional appraisal (Dixon et al., 2017) and emotional generation/expression (Etkin et al., 2011). Recent studies suggest some form of cognitive appraisal involved in the experience of nightmare distress (Gieselmann et al., 2020), but it is unknown which structures contribute to this evaluative process. We hypothesize that the medial prefrontal and anterior cingulate cortices are likely candidates, among other possible structures.

Our results show that retrospective disturbed dreaming frequency was positively associated with ReHo values in the hippocampus. Albeit not a result found in all of our analyses, it is the first result supporting a role for the hippocampus in nightmare production. From a theoretical standpoint, the possible role of the hippocampus — as for the other regions featured in our neurocognitive model of nightmares—is supported by: (1) brain imaging findings that the hippocampus remains active during REM, (2) brain imaging findings suggesting hippocampal alterations in post-traumatic stress disorder, a disorder featuring repetitive nightmares, and (3) a role for the hippocampus in emotion regulation, including in fear conditioning [reviewed in Levin and Nielsen (2007)]. Accordingly, another theoretical model of REM sleep’s role in emotional memory and emotional adaptation also emphasizes hippocampal involvement (Walker and Van Der Helm, 2009). More study is needed to clarify how the hippocampus (and the previously mentioned frontal regions) may contribute to nightmare production. For example, we provide evidence that REM theta activity, a presumed correlate of hippocampal activity, is a marker of frequent nightmares (Marquis et al., 2017).

The findings of associations between nightmares and activity in parietal, occipital and temporal regions would challenge a view that nightmares are only produced by a limbic-prefrontal circuit. While acknowledging that the implication of other regions is likely, the neurocognitive model does not propose a role for these other regions. It is possible that these results are better understood in reference to a ‘differential susceptibility’ model, in which nightmare sufferers are hypothesized to have heightened sensory processing for both positive and negative stimuli (Carr and Nielsen, 2017; Carr et al., 2020a, b). For the moment, no specific brain correlates are posited for this heightened sensory processing that is specific to nightmares, but our results raise the possibility that nightmares are associated with alterations in sensory processing in addition to alterations in emotion processing. Nonetheless, this possibility is speculative, especially given the paucity of literature on the brain correlates of nightmares, as mentioned in the introduction.

Alterations in regions involved in sensory processing may also have clinical relevance. They may help clarify mechanisms underlying the efficacy of Imagery Rehearsal Therapy, a short-term intervention efficient in diminishing nightmare frequency and alleviating nightmare distress (Krakow and Zadra, 2006; Morgenthaler et al., 2018). Despite some recent progress (Rousseau and Belleville, 2018; Gieselmann et al., 2019), the mechanisms underlying interventions specifically targeting nightmares remain unclear. But it has been proposed that linking the brain correlates of nightmares to those of lucid dreaming may give insight into mechanisms underlying reductions in nightmare frequency (Carr et al., 2020b).

At present, no theory of nightmares can account for all the current findings. Despite some limitations that will be described below, the present study builds upon other brain imaging studies of nightmares (Shen et al., 2016; Marquis et al., 2019a) and highlights the need for theoretical developments that would reconcile all the findings. Our study also provides insight into possible common neural substrates (Harvey et al., 2011; Feldker et al., 2017) for nightmares and other psychiatric disorders for which nightmare frequency is elevated (Swart et al., 2013). While beyond the scope of this article, it is worth pointing out that ReHo is altered in a number of such disorders, such as post-traumatic stress disorder (Ke et al., 2017), primary insomnia (Wang et al., 2016) and depression (Iwabuchi et al., 2015). There is also ongoing research on the genetic similarity between nightmares and other disorders (Ollila et al., 2019).

## Limitations and Future Research

It is possible that our focus on disturbed dreaming frequency, rather than on bad dreams or nightmare frequency separately, influenced the results. Since nightmares are considered to be more emotionally and visually intense than bad dreams (Fireman et al., 2014; Robert and Zadra, 2014), they may well have stronger, more easily observable brain correlates. However, our participants did not show much variability in their nightmare frequencies, so combining bad dreams and nightmares optimized the power of our statistical tests.

There are also limitations in our MRI acquisition procedures. Although we matched the sequences from two scanners as closely as possible and made appropriate statistical corrections, we cannot rule out the possibility that our results were impacted. We also cannot rule out the possibility that left-handed participants in our two groups differed in the nature of their brain lateralization profiles—especially with such small samples. Nonetheless, the inclusion of left-handed individuals increases the representativity of our sample and the inclusion of similar proportions of left-handers in each group at least partially mitigates the possibility of significant neural differences. Some characteristics of our study sample may also have affected results. Past research has associated nightmares with neuroticism and/or trait anxiety (Blagrove et al., 2004; Miro and Martinez, 2005) and group comparisons between nightmare recallers and control participants often show differences on these measures (Simor et al., 2012; Carr et al., 2018). Our nightmare participants were not seeking treatment, did not display heightened trait anxiety and were likely less distressed by their nightmares than patients in other clinical studies; thus, our findings may not be generalizable to a broader population of nightmares sufferers.

## Conclusion

This study aimed to document the neural correlates of nightmare-prone individuals using regional homogeneity of the BOLD signal. We compared ReHo values between frequent nightmare recallers and controls. We also correlated ReHo values with nightmare severity variables: NDQ scores, retrospective disturbed dreaming frequency and prospective disturbed dreaming frequency. Our results mainly implicate cortical (parietal, occipital, temporal) areas, as did a previous study from our laboratory (Marquis et al., 2019a). Other results implicating the medial prefrontal cortex, anterior cingulate cortex, and hippocampus (in one analysis), are consistent with a neurocognitive model (Levin and Nielsen, 2007; Nielsen and Levin, 2007). However, the present findings also highlight the limitations of existing pathophysiological models of nightmares as none of these models can easily reconcile all of our results. Thus, more research and theoretical development is needed. While studying nightmare recallers during wakefulness is convenient and useful in demonstrating cross-state alterations in emotional processing or brain structure/function, future research should include brain imaging during sleep along with dream collection. In time, insights gained into the mechanisms underlying nightmare production promise to translate into improvements in clinical practice.

## Data Availability Statement

The datasets presented in this article are not readily available because participants did not agree to this in their signed consent forms. Requests to access secondary results and statistical analyses can be obtained upon request. Requests to access the datasets should be directed to corresponding author.

## Ethics Statement

This study was reviewed and approved by the CIUSSS-NIM Ethics Committee (Hôpital du Sacré-Coeur de Montréal; ID number 2014-1039) and the Ethics Committee Affiliated with the Functional Neuroimaging Unit (ID number CMER-RNQ-15-16-17). Participants provided written informed consent to participate in the study.

## Author Contributions

L-PM: conceptualization, data collection, data analysis, manuscript writing, and revision. S-HJ, CB-C, and MC: data collection and analysis. VD: conceptualization and data analysis. TP: conceptualization, data collection, and analysis. J-PS and JM: conceptualization and funding acquisition. TN: conceptualization, funding acquisition, supervision, manuscript writing, and revision. All authors read and approved the submitted version.

## Conflict of Interest

The authors declare that the research was conducted in the absence of any commercial or financial relationships that could be construed as a potential conflict of interest.

## Footnotes

1 http://www.restfmri.net

2 http://people.cas.sc.edu/rorden/mricron/index.html

## Abbreviations

BA: Brodmann area
BDI-II: Beck Depression Inventory-II
BOLD: blood oxygen level-dependent (signal)
DPABI: data processing assistant for (resting-state) brain imaging
EEG: electroencephalogram/electroencephalographic
FWHM: full-width at half-maximum
fMRI: functional magnetic resonance imaging
KCC: Kendall’s concordance coefficient
MNI: Montreal Neurological Institute
MRI: magnetic resonance imaging
NDQ: nightmare distress questionnaire
NEQ: nightmare experience questionnaire
NM: nightmare
NMD: nightmare distress
PCL-5: posttraumatic stress disorder checklist for DSM-5
ReHo: regional homogeneity
SPECT: single photo emission computerized tomography
SPM: statistical parametric mapping
SD: standard deviation
STAI: state-trait anxiety inventory.
